# The health systems funding platform and World Bank legacy: the gap between rhetoric and reality

**DOI:** 10.1186/1744-8603-9-9

**Published:** 2013-03-06

**Authors:** Scott S Brown, Kasturi Sen, Kristof Decoster

**Affiliations:** 1 School of Population Health, University of Queensland, Herston Rd, Herston, QLD 4006; 2 Wolfson College, University of Oxford, Oxford OX2 6EU, England; 3 Institute of Tropical Medicine, Nationalestraat 155, 2000, Antwerpen, Belgium

**Keywords:** Health systems funding platform, World Bank, Health systems strengthening, Aid effectiveness, Aid coordination

## Abstract

Global health partnerships created to encourage funding efficiencies need to be approached with some caution, with claims for innovation and responsiveness to development needs based on untested assumptions around the potential of some partners to adapt their application, funding and evaluation procedures within these new structures. We examine this in the case of the Health Systems Funding Platform, which despite being set up some three years earlier, has stalled at the point of implementation of its key elements of collaboration. While much of the attention has been centred on the suspension of the Global Fund’s Round 11, and what this might mean for health systems strengthening and the Platform more broadly, we argue that inadequate scrutiny has been made of the World Bank’s contribution to this partnership, which might have been reasonably anticipated based on an historical analysis of development perspectives. Given the tensions being created by the apparent vulnerability of the health systems strengthening agenda, and the increasing rhetoric around the need for greater harmonization in development assistance, an examination of the positioning of the World Bank in this context is vital.

## Background

Limited progress to date on the achievement of the health-related Millennium Development Goals (MDGs) has led to the realization that selective approaches to health and development have been both ineffective and problematic [1-4]. This realization has driven the renewal of the health systems strengthening (HSS) concept, which is now prominent in the wider aid effectiveness agenda [1,5-8]. Indicative of its ascendency is the level of resources and promotion it is now afforded - particularly by a number of high profile and well-resourced multilateral development agencies. These include the Global Fund to Fight AIDS, Tuberculosis and Malaria (Global Fund), the GAVI Alliance (GAVI), and the World Bank (the Bank), which have all stepped up their HSS funding in recent years [8], as well as amplified their advocacy for its importance in achieving sustainable population health outcomes and poverty reduction [5-7].

A product of this increased focus has been the recent formation of the Health Systems Funding Platform (Platform); a funding collaboration between the Global Fund, GAVI and the World Bank, facilitated by the World Health Organization (WHO). The Platform was established in 2009 on the recommendation of the High Level Taskforce on Innovative International Financing for Health Systems (TIIFHS), and is intended to act as a mechanism to accelerate progress towards the MDGs. Specifically, it aims to: “… coordinate, mobilize, streamline and channel the flow of new and existing international resources to support [HSS through] national health strategies” [9].

To improve efficiency – and to subsequently enhance aid effectiveness - GAVI and the Global Fund trialled a common application form for new funding, with a view to developing a similar process for countries seeking funding through a Joint Assessment of National Strategies (JANS). In addition, all three partners propose to harmonize their monitoring and evaluation practices where a common reporting system and set of indicators can be agreed between Platform partners and recipient country [9].

### Donor ‘collective’: advantage or impediment?

The expansion into HSS is relatively new for GAVI and the Global Fund; an extension of their rounds of targeted interventions delivered in the space of immunization and the three diseases respectively. The impetus behind their move appears to be the growing recognition that early gains achieved under their specific mandates cannot be extended without overcoming broader health system obstacles [2,5-7,10,11]. Another view is that all three agencies perhaps recognise the need to better align themselves with changing donor priorities, where HSS may be seen as the way forward [10,11]. Irrespective of the motivation behind their move, this “mandate to mingle” has caused concern and anxiety among some development stakeholders – perhaps none more so than civil society organizations [12,13].

The cause for concern is well understood: neither GAVI nor the Global Fund has the necessary HSS technical expertise or the in-country presence to effectively monitor and assist countries in HSS policy development and implementation [13,14]. This means that, by default, the World Bank – which has a self-proclaimed comparative advantage in this space [7] – is best positioned to oversee and lead on the Platform’s in-country policy role [15].

The Bank’s leadership in this regard – whether implicit or explicit – should be relatively inconsequential, as agreement between Platform partners to employ the Paris Declaration and International Health Partnership Plus (IHP+) aid effectiveness principles should provide consistency and predictability around the Platform’s approach [9]. It should also assure donors that their investments are being used efficiently and effectively – in line with agreed development principles.

We would argue however that any sense of assurance granted by the Platform’s aid effectiveness rhetoric should be accepted with some caution, particularly if one subscribes to the idea that ‘past behaviour is often the best predictor of future behaviour’. The interruption to new HSS activities with the Global Fund’s suspension of Round 11 is very pertinent in this regard, demonstrating the ease with which it can contain HSS support in favour of its selective three diseases mandate. But while current attention may be focused on the Global Fund, this paper aims to contribute to the rather thin critique of the World Bank’s involvement in the Health Systems Funding Platform. Specifically, it examines the Bank’s inability to comply with the aid effectiveness principles adopted by the Platform to support its claim on innovative HSS funding. In addition, it illustrates the Bank’s apparent reluctance to harmonize its funding processes with other Platform partners, casting further doubt over the Platform’s ability to fulfil its mandate. The intention is to stimulate greater discussion around the appropriateness and merit of having the World Bank play such an influential, and more specifically a leadership, role in the Platform. Closer scrutiny and open debate is essential if effective, timely and sustained outcomes are to result from the global health community’s increased commitment to HSS [16].

## Methods

This research uses a case-study design [17] to examine the practices and impact of World Bank development policy intervention in borrower countries over almost two decades. It also examines the available literature on the Health Systems Funding Platform to identify implications between the World Bank’s activity and the Platform’s mandate. Sources published from 1993 were included in order to inform the historical analysis that supports much of this essay’s argument.

To enhance its rigor and validity [18], the research design uses two qualitative research methods. The first is a review of the available literature, drawing on peer reviewed papers, data sets and grey literature [19]. These were sourced using Google Scholar and Medline search engines between 8 February 2011 and 28 January 2013. Keywords and titles relating to ‘World Bank’ – coupled with ‘health’, ‘development’, ‘conditionality’, ‘policy’; and ‘Health Systems Funding Platform’; ‘aid effectiveness’; and ‘health systems strengthening’, were used to generate results. Organizational websites including: The World Bank, the Global Fund, the GAVI Alliance, the World Health Organization, the OECD, and the IHP+ were also used to source information as late as 11 March 2012.

Secondly, participant observation by the authors and their colleagues in processes related to the Platform and to the World Bank were drawn on (analysis and evaluation of funding proposals, membership of relevant boards, advisory groups and committees, related research) [20] between 2010 and 2012. Thematic analysis was conducted manually by the lead author. Differences were discussed and agreement was reached between all authors on the final analysis structure.

## Discussion

### A “wind of change” at the bank

Prior to its engagement in the Platform, the World Bank clearly demonstrates a rhetorical preference for a more comprehensive, ‘country-owned’ approach to health sector investment. This symbolic change can be traced back most notably to its 1993 ‘World Development Report: Investing in Health’, where its discourse clearly takes on a sentiment of HSS through capacity building and partnership: “The efficiency for which aid for health is spent depends critically on building local capacity to plan and manage health systems” [21].

The Bank attempted, to some degree, to implement its new ideology; evident in the rollout of its Sector Investment Programs (SIPs) [22], followed by the Sector Wide Approach (SWAp) [23]. The principles promoted under both SIPs and SWAps were of government “ownership” and “partnership”, which complemented the argument for a more comprehensive development strategy, and of building local capacity as a means of achieving sustainable development outcomes. Through SWAps, the Bank described its evolving directional change as “…an approach to locally-owned programs for a coherent sector in a comprehensive and coordinated manner, [with an emphasis on] moving towards the use of country systems” [23].

It was the perception of past development failures that helped steer the Bank’s approach towards this new paradigm. From the days of ‘structural adjustment’, “dominant lessons” were learnt by the Bank through its own research on aid effectiveness, in which it conceded that “…donors can advise on and support, but cannot buy or induce, economic [and state] reforms. Thus government willingness to reform – where possible, with broad popular support – is essential to popular programs” [24]. The Bank also learnt that “Empirical studies emphasize that policy changes are driven primarily by the domestic political economy, not by foreign assistance or policy-based lending” [24]. Embracing the country ownership/partnership paradigm was clearly regarded by the Bank as the necessary alternative to the hierarchical means synonymous with its policy-based lending and conditionality approach [25-33].

The proclaimed adoption of this philosophical shift was made particularly explicit through a series of declarations announcing a “wind of change” in the Bank’s development practices. Former World Bank President, James Wolfensohn, summed up this directional change in 1999 stating that:

What is new is an attempt to view our efforts within a long-term, holistic and strategic approach… Such development should, in our judgement, be a participatory process, as transparent and as accountable as possible within the political climate prevailing in each country… It is a holistic and strategic approach to development based on country ownership and partnership… It is also a commitment to expand partnerships, transparency, and accountability under the leadership of government [34].

Following a clear espousal of aid effectiveness principles through the 1990s, the Bank continues to promote these through formal policy positions and advocacy [35]. Despite the apparent adoption of a new approach however, discrepancies between its rhetoric and its actual practices have become evident.

### Implementing the aid effectiveness principles: rhetoric ≠ reality

The Bank’s track record for genuinely implementing country ‘owned’ and ‘locally-driven’ development policies, to date, is rather poor [25-32,36-44]. Conversely, its reputation for ‘strong-arming’ recipient governments into state and economic reform through coercive, hierarchical governance has become notorious with its policy-based lending/conditionality approach. This development strategy has been dominant at the Bank since the days of “structural adjustment” (early 1980s), with evidence suggesting that it remains so long after [45-48].

Research conducted by the World Bank during its “Conditionality Review” (2006) demonstrated the opinion of developing country governments regarding the Bank adequately respecting country ownership. Of those interviewed:

• Almost half – 49% - agreed that “World Bank supported policy programs introduce new elements that are not part of my country’s medium and long term development strategy”

• More than half – 56% - thought that “my government’s original policy program was significantly modified in negotiations with the World Bank”

• Three quarters – 77% - agreed that “World Bank multi-sector operations significantly increase the number of policy actions my country must deliver to obtain financial support” [32].

These sentiments were also reflected in the research conducted by Eurodad, which found evidence of the Bank actually increasing the number of policy-based lending conditions on borrowing countries between 2002 and 2005 [45] - as opposed to decreasing them as it had claimed [46]. Eurodad examined the World Bank loan documents of 20 low-income borrower countries, and found that of these, 14 of them had more than 50 conditions attached to each of their loans, and 3 out of 20 had more than 100 conditions attached [45,47,48]. This saw the average number of conditions per loan amongst these 20 countries rise from 48 in 2002 to 67 in 2005 [45] – six years after the Bank openly acknowledges the failure of conditionality [34].

The foreign policy influence evident here has also been found in Pakistan, where Bank officials conceded that the Poverty Reduction Support Credit (PRSC) matrix – a document prepared by the World Bank, not the developing country government – is effectively the document that spells out how Pakistan’s Poverty Reduction Strategy Paper (PRSP) will be rolled out [32]. Similarly in Afghanistan, Roberts (2009) describes the control the Bank-IMF had over its PRSP, seriously questioning how such a document can be truly ‘country-owned’ given it is “shape[d]” by the Bank-IMF into what it requires for approval [43].

Tuozzo (2004) also describes the coercive influence that the World Bank’s policy intervention has had in Argentina, finding that “Bank policy research is a discursive or ideational form of power that helps to set and sustain development agendas, some of which might not be the most adequate or beneficial to a borrowing country” [29].

Moreover, more than 600 tribunal attendees at the Independent People’s Tribunal in India heard hundreds of testimonies and formal depositions from World Bank-project affected stakeholders from across the country. “Almost all the testimonies overwhelmingly pointed to the Bank’s undue (and often negative) influence in shaping India’s national policies”, and that ‘the negative impact of the Bank’s policy and project interventions were apparent in every major sector of the economy and society’ [49].

Despite evidence demonstrating almost two decades of undue policy influence, the World Bank continues to advocate for country-owned and aligned development approaches, giving it high priority in its 2007 Health, Nutrition and Population (HNP) Strategy: “Country-driven and country-owned programs are the key to good HNP results on the ground”, ‘and the challenges that need to be faced in regard to development assistance for health (DAH) include’ “…the need to align and harmonize global partners’ activities with country needs to prevent duplication, economic distortions – and excessive administrative costs – ensuring country-owned and country-led DAH” [7].

This rhetoric clearly remains prominent at the Bank today; being used currently to describe the Platform’s guiding framework for HSS activities: the Platform “…is based on the principles of the International Health Partnership Plus (IHP+), in line with the Paris Declaration on Aid Effectiveness, to promote: national ownership; alignment with national systems; harmonization between agencies; managing for results; and mutual accountability among partners, donors and countries” [9].

With such evident discrepancies occurring between the Bank’s aid effectiveness rhetoric and its actual practices – and given the length of time over which they have been occurring, questions must be asked about the merit and appropriateness of having the Bank play such an influential role in the Platform’s in-country HSS activities.

### Non-compliance with aid effectiveness principles: a practical necessity?

While the trend in coercion and undue influence by the Bank has been well documented over time [25-32,36-44], the dichotomy between its behaviour and its policy rhetoric, and the potential reasoning behind it, have been explored less thoroughly.

Turning briefly to the latter, it is understood that in some instances, developing country governments are perceived by donors as having weak capacity, inadequate accountability and compromised integrity [50-54]. ‘Coordination led by recipient country governments can often be traced to weaknesses in policy and planning processes, and inadequacies in the institutional arrangements established to coordinate aid’ [50].

Giri et. al.’s (2013) recent study into the Government of Nepal’s aid coordinating capabilities highlights this issue:

Despite its commitment to coordinate and control development assistance to the health sector, and its leadership position of the Sector Wide Approach, complete knowledge and effective coordination of all international contributions remains a challenge and is hampered by issues within the government as well as among External Development Partners and International Non-Governmental Organizations [55].

Conveying the familiar result of perceived recipient government weaknesses, a Swedish evaluation of its aid to Bangladesh, including the World Bank/cofinancier FPHP, found that: ‘…it is rather self-evident that the government’s coordinating role falls victim to interests of efficiency. The nature of the programs as gigantic by-pass operations, set to avoid the negative influence of the bureaucracy, tends to be perpetuated’ [50,56].

In addition to interests of efficiency, donors will often bypass government systems in an attempt to avoid or minimise potential corruption and misappropriation [51-54]. Haiti, with its influx of development and humanitarian aid in recent years, provides an example of where concerns over both government capability and corruption have had this effect [57]. The assertion by some that billions of dollars in aid – a proportion of which has been channelled through government infrastructure - has done little to improve the lives of Haitians, or to enhance the country’s governance and institutional capacity, somewhat supports donor decisions to explore alternatives to government-led aid coordination [57].

#### The politics of aid coordination

As well as matters of capability and integrity, the growing political connotations associated with foreign aid (since 2001 in particular) appear to also shape a donor’s decision to use or bypass recipient government systems:

Stakeholders are well aware of the power, influence and leverage which aid coordination confers, an awareness which colours the desire of some stakeholders to lead aid coordination processes, and conditions the extent and manner by which others wish to be involved [50].

USAID’s decision to channel tens of billions of dollars through the Egyptian and Pakistani governments, for example – both of which have been plagued for years by proclamations of corruption and financial mismanagement [58,59], illustrates one instance of this. The US has seemingly chosen to trade-off matters of efficiency and effectiveness for the advancement of a number of its foreign policy objectives (largely regional stability and support for the ‘war on terror’) [60].

The assertion that US foreign policy objectives colours the extent to which the US applies its development aid control measures has considerable implications for the World Bank and its clients. It has been well argued that the US often has disproportionate influence over the World Bank’s fund disbursement and conditionality decisions [61-63]. Kilby (2009) finds specifically that countries that are ‘not friendly’ to the US (those that do not make concessions to the US position in important UN votes, for example) have their World Bank loan conditions more strictly enforced than those that are favourable to US positions [63]. This notion provides further consideration to why the World Bank may exert – or conversely relax – control over aid coordination in recipient countries.

### Donor control *for* effectiveness: a paradox

Irrespective of the motive behind a donor’s decision to control aid, or to exert undue influence over a country’s development policies, ample evidence demonstrates its categorical ineffectiveness [64]. Thus using it for *greater* effectiveness is both paradoxical and problematic.

Acknowledgement of this very notion is what has helped drive the advocacy for aid effectiveness principles by the World Bank (and GAVI and the Global Fund for that matter), and hence why they have used this philosophy to drive the Platform’s claim on innovative HSS funding.

However, as this paper has demonstrated throughout, despite promoting one development philosophy, the World Bank in reality does something quite different. Its willingness to disregard aid effectiveness principles in favour of its hierarchical ‘business as usual’ approach to development, poses some real concerns for the Platform. Most importantly, it raises serious questions around its appositeness for the task.

### Partnership tensions

As well as failing to genuinely comply with the aid effectiveness principles adopted by the Platform, tensions between the Bank and other Platform partners are becoming apparent. The World Bank convened Independent Evaluation Group (2006) highlights this issue, finding that:

GRPPs [Global and Regional Partnership Programs] with shared governance arrangements present a number of challenges for the Bank’s traditional country-based business model. First, they challenge the Bank’s traditional financial and managerial accountability mechanisms. Their legal and governance arrangements do not always confer sufficient clarity on how the collective responsibility for the programs works in practice and may set limits on the Bank’s authority that are not consistent with its accountability [65].

The Bank’s reluctance to harmonize its HSS application and funding processes with those of its Platform partners is seemingly indicative of these issues [11].

Adding to the structural difficulties are the relationship tensions becoming evident between Bank and Global Fund operational staff:

Both Global Fund and Bank operational staff would prefer engagement in the context of their own organization’s business model. They generally viewed the comparative advantages of the other organization in terms of what the other could contribute to its own method of operation [66].

Partnership impediments are reinforced by the fact that there is no formal agreement or memorandum of understanding between the Bank and the Global Fund in terms of working together at country level - nor have there been any written directives or guidelines issued to staff in either organization to support this [66].

These impediments, combined with the Bank’s inability to implement the aid effectiveness principles that it so openly adopts, cast shadows over the Platform’s ability to deliver effectively on its HSS mandate. Notwithstanding, this partnership still reflects a paradigmatic shift in the way multilateral agencies harmonize practices in a bid for better aid coordination, and its concept should be welcomed and promoted. But unless due caution and much needed debate is afforded to the evident discrepancies between the Bank’s rhetoric and its practice, and unless it is able to better cooperate with its partners at country-level, the Platform and all that it intends to achieve risks resulting in another development intervention failure.

## Summary

While international attention is currently focussed on the Global Fund’s Round 11 suspension, and what this might mean for HSS activity, little critique has been offered of the World Bank’s ambivalence around ‘country-ownership’ and ‘partnership’ and its limited procedural flexibility within the Platform.

Despite recognition of its failure to achieve adequate health and development outcomes using its traditional policy-control/excessive influence approach [46], and its subsequent rhetorical adoption of the Paris Declaration and IHP+ principles [7], evidence demonstrates that hierarchical governance persists as the dominant development practice at the Bank [25-33,43,44].

Reasons for this occurrence can, in many instances, be understood (even if not agreed with) [50-54]. However, this does not displace the fact that building a partnership – with such sizeable resources and expectations – on a platform of principles which are not complied with by, arguably, its most important member, poses a serious risk to the integrity of the partnership, and more importantly, to the outcomes it promises to deliver. The lack of critique to date on the World Bank’s role is perhaps indicative of a more systemic issue; that insufficient consideration is being given to partnerships created for political and bureaucratic convenience. This paper calls to redress this issue, and argues for a critical approach to global health partnerships through increased discussion and debate, before too many resources and a growing level of expectation result in another development intervention becoming a costly and ineffective burden.

## Competing interests

The authors declare no competing interests.

## Authors’ contributions

SB conducted the literature research, and KS and KD provided input derived from their own experience and that of colleagues. All authors participated in the thematic analysis. SB developed the first draft and led on its overall development. KS and KD provided further input and edits throughout the revision process. All authors read and approved the final manuscript.
